# Successful living donor liver transplant in a very small child

**DOI:** 10.4103/0019-5049.68392

**Published:** 2010

**Authors:** Vijay Kumar, Raman Raina

**Affiliations:** Department of Anaesthesiology and Pain Relief, Indraprastha Apollo Hospitals, New Delhi - 110 076, India

**Keywords:** Living donor liver transplantation, paediatric, small child, congenital biliary atresia

## Abstract

Liver transplantation in small children poses perioperative challenges that are different from those seen in adults. We present our successful anaesthetic experience in a 7-month-old infant who has been the youngest case of successful living donor liver transplant performed in our institution till the day this article was being prepared.

## INTRODUCTION

Traditionally, small-sized and younger children had a worse prognosis after liver transplantation, but now these outcomes are changing with increasing experience.[1]

Most available literatures in paediatric liver transplantation describe an infant as a small child without any defined subdivision. It may be either less than or more than 10 kg body weight as the surgical anastomosis in <10 kg child is usually considered difficult. Small infants continued to have poor outcomes because of the technical challenges of creating and maintaining patent vascular anastomoses and waitlist mortality caused by the extreme shortage of appropriately sized small donors.[2]

The first successful paediatric orthoptic liver transplantation was performed in 1967 and the first living donor liver transplant (LDLT) was attempted in 1988. Advances in recent years have made LDLT an established treatment in children.

## CASE REPORT

Our patient, a case of congenital hyperbilirubinaemia, had undergone the Kasai procedure at 5 months of age. About 2 months later, the child developed fever and hyponatraemic seizures for which he was treated conservatively at a local hospital and, later, was referred to our hospital for further evaluation of a possible liver transplant. The child, on admission, was conscious, haemodynamically stable and had a total body weight of 7.5 kg. Laboratory investigations are shown in Table 1.

**Table 1:** Investigations report at admission

Hb	9.8 g/dl
Platelets	85,000/mm^3^
Blood urea	10 mg/dl
Serum creatinine	0.2 mg/dl
Serum Mg.	2.1 mg/dl
Na	136 meq/L
K	2.6 meq/L
Serum bilirubin	48.3 mg/dl
Direct bilirubin	35.0 mg/dl
SGOT	258 IU/L
SGPT	42 IU/L
Alkaline phosphatase	406 U/L
SGGTP	159 U/L
Albumin	3 g/dl
PT	25.6 s
INR	2
Viral serology	Negative
ECG	Normal
Chest X-ray	Normal
Ultrasound abdomen	Portal vein thrombosis with reversal of flow
ABG	pH 7.33/PCO_2_ 33.3/pO_2_ 229/HCO_3_ 16.9

The operating room was pre-warmed to 25°C and the table was equipped with a warming mattress. We used the Bair hugger warming blanket device, a fluid warmer, and covered the patient’s limbs with a soft cotton roll to prevent hypothermia. The child was anaesthetized with Fentanyl 2 mcg/kg, Thiopentone 5 mg/kg and Atracurium 0.5 mg/kg body weight and was intubated with an oral uncuffed endotracheal tube of size 4.0 (Portex, UK). Anaesthesia was maintained with isoflurane in air–oxygen mixture, Fentanyl (2 mcg/kg/h) and Atracurium (0.5 mg/kg/h) infusion. Intermittent positive-pressure ventilation was carried out using the pressure-controlled mode.

The right radial artery was cannulated (24 G) and a triple-lumen central line (4.5 F, 6 cm) was placed in the right internal jugular vein to measure invasive BP and central venous pressure (CVP), respectively. An intravenous 18 G cannula was also put in the right cubital vein.

Nasopharyngealtemperature, invasive arterial blood pressure (ABP), electrocardiogram (ECG), peripheral oxygen saturation (SPO_2_), end-tidal carbon dioxide (ETCO_2_) tension, hourly urinary output and CVP were monitored continuously. Haemoglobin (Hb), haematocrit, electrolytes, blood sugar, prothrombin time (PT), INR, activated partial thromboplastin time (aPTT) and arterial blood gases (ABG) analyses were performed every 60 min during the hepatectomy phase and 30 min during the anhepatic and post-reperfusion phases. Coagulopathy was monitored with computerized thromboelastography (TEG). The dissection phase, in addition to blood loss and third-space loss, was marked by the development of reduced urine output and acidosis, requiring frusemide and sodium bicarbonate infusion. Sodium bicarbonate needed to be continued up to the post-reperfusion phase when the graft itself started correcting the acidosis.

The child received a segment II and III hepatic graft weighing 308 grams from the left lobe of his father’s liver.

There was clinically no significant haemodynamic change at the inferior vena cava clamping. However, at reperfusion, there was a fall in blood pressure, which responded to a small dose of phenylephrine. The total amount of fluid required was 5% dextrose with 0.18 N saline 300 ml; 5% albumin 250 ml; plasma (single donor unit) two half units; modified fluid gelatin (Gelofusine, B.Braun) 200 ml; packed red cells three half units; normal saline 200 ml. After hepatic artery anastomosis, Doppler ultrasonography confirmed good flow in the vessels. Finally, a rouxen Y hepaticojujenostomy was performed followed by closure of the surgical wound. Total anaesthesia time was just over 14 hours. The child was extubated 36 hours later in the intensive care unit. However, the child was re-explored twice, once for portal vein thrombosis for which a cava portal anastomosis was done and the second time 2 days later for bleeding from the graft portal vein. The child continued to improve afterwards and was finally discharged in a satisfactory condition about 4 weeks after surgery with good liver functions [Table 2]. The child is alive and is doing well till date.

**Table 2:** Liver functions at discharge from hospital

Serum bilirubin	0.76 mg/dl
Direct bilirubin	0.42 mg/dl
AST	28 IU/L
ALT	29 IU/L
PT	12.1 s
INR	1.0

## DISCUSSION

The common indications for hepatic transplantation in the paediatric population clearly differ from those in adults [Table 3]. Pre-operatively, nutritional supplementation is essential and nutritional assessment may be difficult as an increase in weight may reflect worsening organomegaly or ascites rather than true growth.[3]

**Table 3:** Common indications of liver transplantation

Cholestatic conditions, e.g. EHBA
Fulminant liver failure
Metabolic liver disease, e.g. Wilson’s disease, Crigler-Najjar
syndrome
Miscellaneous
Cryptogenic cirrhosis
Hepatoblastoma
Liver tumours
Drug overdose or toxicity
Re-transplant

Hepatectomy of the native liver can pose numerous challenges to the anaesthetist. Prior operation for biliary atresia or multiple episodes of spontaneous bacterial peritonitis lead to extensive adhesions, increasing the risk of intestinal perforation and bleeding at hepatectomy.[4] However, our case did not bleed much even in the presence of adhesions due to slow but meticulous surgery.

Children tolerate clamping of the vena cava better than adults, often with no significant change in systolic blood pressure despite a significant drop in pre-load and mean pulmonary artery pressure. Veno-venous bypass is not used in children of <10–15 kg because of the difficulty in maintaining adequate flow through the small cannulas.[5]

Clamping of the portal vein during portal vein thrombectomy, resulting in gut ischaemia, aggravated lactic acidosis, requiring sodium bicarbonate. Blood, blood products and fluids were used according to the need for correction of coagulopathy (INR 1.5–2.5, platelet count >30,000), to maintain Hb between 8 and 10 gm/100 ml and a CVP of 8–10 mmHg. Among the paediatric patients, LDLT is associated with longer operation times and higher RBC and fluid requirements than cadveric donor transplants.[6]

During the anhepatic phase, the patient was optimized regarding serum electrolytes (especially potassium and ionized calcium), acid–base status, coagulability and Hb concentration. Reperfusion syndrome is the most stressful period and may be associated with hypotension and cardiac arrhythmias.

After liver transplantation in children, a 50% decrease in the plasma concentrations of both protein C and antithrombin III and a 10-fold increase in plasminogen-activator inhibitor in the immediate post-operative period leads to an increased risk of thrombosis.[7] This may have contributed to the post-operative portal vein thrombosis in our case.
